# Context-dependency of monarch butterfly (*Danaus plexippus*) egg abundance on milkweeds (*Asclepias*)

**DOI:** 10.1371/journal.pone.0336242

**Published:** 2025-11-04

**Authors:** Katie Harris, Timothy M. Reinbott, Terry Woods, Jared M. Brabant, Grant Harris, Deborah L. Finke

**Affiliations:** 1 Division of Plant Science & Technology, University of Missouri, Columbia, Missouri, United States of America; 2 Missouri Agriculture Experiment Station, College of Agriculture, Food and Natural Resources, University of Missouri, Columbia, Missouri, United States of America; PMAS Arid Agriculture University: PMAS-Arid Agriculture University Rawalpindi, PAKISTAN

## Abstract

North American monarch (*Danaus plexippus*) populations have experienced sharp declines. Loss of milkweed is among the major drivers of this decline. Our objective is to identify factors that influence milkweed habitat quality for monarchs to inform habitat reconstruction efforts. We measured the response of monarch egg abundance to milkweed patch characteristics (milkweed species identity and co-occurring insects) and features of the surrounding landscape (wildflower nectar plants and land use context). From May through September 2019 and 2021, we assessed the abundance of naturally occurring monarch eggs, lady beetles (predators), and aphids (competitors) on swamp milkweed (*Asclepias incarnata*) and common milkweed (*A. syriaca*) plants established in a diverse matrix of wildflower nectar plants or monoculture of fescue grass and located in open row crop habitats or abutting wooded edges. We found that local patch characteristics had the largest effects on monarch egg abundance. Egg abundance differed across milkweed species, but the milkweed species with the highest monarch egg abundance switched from common milkweed in 2019 to swamp milkweed in 2021. We also found that monarch egg abundance was positively associated with oleander aphid (*Aphis nerii*) abundance on both milkweed species, despite significantly higher aphid abundance on swamp milkweed than common milkweed. Lady beetles exhibited a numerical response to oleander aphid prey, but there was no evidence that greater numbers of these generalist predators negatively affected monarch eggs. Landscape features also influenced monarch egg abundance, but the effects varied across milkweed species and years. In 2019, egg abundance was higher on swamp milkweed near trees than in open crop fields, while proximity to wildflower nectar plants increased egg abundance on common milkweed; landscape features did not directly affect egg abundance in 2021. Our results highlight the important role of environmental context in mediating the conservation value of milkweed plantings for monarchs.

## Introduction

The monarch butterfly (*Danaus plexippus*) is an iconic insect pollinator that requires conservation action in North America to preserve its unique transcontinental migration. Monarch butterfly populations are in sharp decline, with the largest of these populations, the eastern population located east of the Rocky Mountains, experiencing an 80% drop in abundance since the 1990’s [1,2]. Several factors are implicated in this decline, but the loss of milkweed plants from agricultural environments is a major contributor [3].

Adult monarch butterflies require larval hosts plants in the genus *Asclepias* (also known as milkweed) and their close relatives to complete their lifecycle [4]. Availability of milkweeds in the Midwest region of the U.S., a crucial breeding area for eastern monarch population, declined sharply over the past two decades, particularly in agricultural landscapes [3,5]. For example, a 68 percent loss in milkweed availability was documented in Illinois croplands between 1997 and 2017 [5]. In response to declining host plant resources, there is a societal push towards planting more milkweed [6]. However, milkweed plants are part of a complex environment and their use by monarchs is likely affected by local patch characteristics, including milkweed species identity and co-occurring insects, and features of the surrounding landscape, such as access to wildflower nectar plants and land use context [7,8]. The challenge is to design monarch habitats that optimize access to required plant resources while minimizing potential negative interactions [5].

There are 77 species of milkweed (*Asclepias*) found in the United States, and monarch oviposition preference and larval performance vary across milkweed species [9–13]. When offered a choice in a controlled environment, monarch butterflies preferred to oviposit on swamp milkweed (*A. incarnata*) over nine other species [9,11,13]. Monarch larvae also had the highest survival and growth rates on swamp milkweed in no-choice studies [11]. However, in a complex real-world environment, uncontrolled factors are likely to confound the effects of milkweed species on monarch oviposition and egg survival. For example, monarch butterflies infected with the protozoan ectoparasite *Ophryocystis elektroscirrha* (OE) prioritize milkweed species with high concentrations of anti-parasitic foliar cardenolides for oviposition, rather than nutritionally favored plants like swamp milkweed [14,15].

Less well-studied is the effect of the co-occurring insect community, including natural enemies and competitor insects, on monarch preference for and performance on different species of milkweed. Competitors, including the phloem-feeding oleander aphid (*Aphis nerii*), commonly co-occur on plants with monarch eggs and larvae. Oleander aphids colonize milkweed plants in late summer when adult monarchs are ovipositing and quickly reach high densities [16]. Aphids feeding on common milkweed (*A. syriaca*) benefit larval monarchs by interfering with the plant’s induction of defenses against chewing feeding herbivores [17]. However, feeding by aphids also reduces foliar cardenolide concentrations, thus limiting the ability of larval monarchs to fight diseases such as the protozoan ectoparasite OE [18].

Generalist insect predators are also common inhabitants of milkweed plants. Only 20% of monarch eggs survive to hatching with fewer than 2% of these larvae successfully developing into third instars [19]. Predation by other insects is thought to be a large driver of this low survival rate [19]. Monarch larvae and eggs are susceptible to predation by a wide range of insects including earwigs (Forficulidae), tree crickets (Gryllidae), spined soldier bugs (*Podisus maculiventris*), lady beetles (Coccinellidae) and lacewing larvae (Chrysopidae) [20–22]. Few studies have examined how predation risk varies across milkweed species [23].

In addition to competing with monarchs, aphids may influence monarch susceptibility to generalist predators. The presence of aphids increases the abundance of generalist predators on milkweed [24] and thus may indirectly enhance predation pressure on monarch eggs and larvae. An ecological interaction called apparent competition. Conversely, aphids can benefit monarchs by indirectly reducing predation pressure by serving as alternative prey for generalist predators. For example, the multicolored Asian lady beetle (*Harmonia axyridis*) is more likely to consume aphids than larval monarchs when offered together [25]. Although much is known about the effects of community interactions on larval monarchs, the effects of predators and competitors on monarch egg abundance are understudied.

At the landscape level, the surrounding plant community, including the presence of non-larval-host wildflowers, can affect monarch use of milkweed plants. As a critical source of flight fuel for adult females [26], increased proximity of wildflowers may encourage monarch oviposition on milkweeds. In fact, monarch egg abundance was 22% higher on milkweed plants located in mixed-species plantings than in a monoculture of milkweed [27]. Alternatively, milkweed found within a wildflower matrix may be more difficult for monarchs to locate [28]. Habitat complexity through increased plant diversity can interfere with volatile detection and visual searching and negatively affect host plant location by insects [29,30].

Land use of the surrounding landscape can also influence monarch egg abundance [8,31]. Milkweed patches embedded within an intensively cropped environment may experience greater pesticide exposure and overall lower insect diversity [3]. Alternatively, adjacent trees may provide adult butterflies with resting sites and protection from high temperatures [32]. However, natural enemies can also be more prevalent at habitat edges near wooded areas, potentially enhancing the rate of predation [33]. More work is needed to understand the potential importance of landscape context in determining habitat quality for monarchs.

Our primary goal is to understand how local patch features and landscape context interact to determine monarch egg abundance on milkweed plants in an agricultural environment. To accomplish this, we established field plots that varied in milkweed species identity, presence of wildflowers and land use context. We measured the abundance of naturally occurring monarch eggs, predators and competitors found on milkweed weekly throughout the growing season. We used structural equation modeling to tease apart the direct and indirect effects of patch- and landscape-level characteristics on the abundance of monarch eggs to determine which aspects of the environment are most influential in supporting monarchs.

## Materials and methods

### Study area

Experimental plots were established at South Farm Research Center (Central Missouri Research, Extension and Education Center, Columbia MO) in the summer of 2018. No permits were required on university property. Plots were established into marginal grass habitats, either old fields or the grassy field borders surrounding conventionally managed soybean (*Glycine max*) and maize (*Zea mays*) crops. One plot consisted of fourteen milkweed plants, either swamp milkweed (*A. incarnata*) or common milkweed (*A. syriaca*). The milkweed species treatment was crossed with a plant community treatment: milkweed located within a diverse mix of wildflower species (S1 Appendix) or milkweed surrounded by a monoculture of tall fescue grass (*Festuca arundinacea*) (S2 Appendix). These four treatment combinations constituted one experimental block (S3 Appendix). Each of the four plots in an experimental block was 6m × 6m with a 3m grass buffer between plots. Ten blocks were established, five located in old fields and grassy field margins within 5m of a wooded tree line (S4 Appendix) and five in the grassy margins of conventionally managed soybean and maize crops and at least 150m from the nearest tree line (S5 Appendix). Blocks were separated by at least 50m and were on average 438.6 ± 28.9m apart (mean ± SE).

All milkweed and wildflower plants were grown from locally sourced seed (Hamilton Native Outpost^©^, Elk Creek MO) in 72-count greenhouse flats during spring 2018. Plot locations were prepared for planting with a glyphosate treatment in June 2018 followed by tillage. Milkweed and wildflower plants were planted in the field as seedling plugs in July and August 2018, respectively. Tall fescue was sown directly into the soil as seed in September 2018. Ten wildflower species were used (S6 Appendix), with 28 seedling plugs of each species per plot that received wildflowers (280 total plants). Species were chosen for use based on bloom phenology, attractiveness to monarchs and hardiness to local weather and climate. Individual wildflower seedlings were distributed randomly across the plot using a random number generator (S7 Appendix). The arrangement of wildflower species was the same for plots within the same block but differed across blocks. Field plots were reassessed and weeded in late April and early May 2019 to ensure plot establishment. Missing plants were replaced with new seedlings at this time. Plots were weeded in future years, but missing plants were not replaced.

### Sampling

We conducted visual sampling of milkweed plants weekly from May through September of 2019 and 2021. (Data for 2020 are unavailable due to university closures during the COVID-19 pandemic.) Milkweed plants were sampled between the hours of 6am and 1 pm. The order of sampling blocks and plots within blocks was randomized to avoid time of day effects. One stem from each of the 14 milkweed plants in a treatment plot was thoroughly searched for insects, including the stems, leaves and flowers (if present). We recorded the number of monarch eggs and other co-occurring insects, primarily aphids and lady beetles (Coccinellidae larvae and adults). When the number of aphids was too high to reasonably count individuals, we estimated aphid abundance by counting groups of 10 estimated aphids. It is important to note that because this was an open field study the number of monarch eggs observed on plants was a function of both the oviposition decision of the adult female and egg loss due to predation or other abiotic and biotic factors. Milkweed plant height was measured weekly for 3 randomly selected stems in each plot for one year and values were averaged per plot.

### Comparison of monarch egg and aphid abundance across milkweed species

We compared the cumulative number of monarch eggs found on common milkweed and swamp milkweed for 2019 and 2021 using a negative binomial generalized linear model (GLM) for count data that exhibits overdispersion. Seasonal egg abundance in 2019 was bimodal, with an early season peak corresponding to the arrival of migrants and a late-summer peak associated with breeding populations. Therefore, we conducted follow-up negative binomial generalized linear model (GLM) tests comparing egg abundance on common milkweed and swamp milkweed for early season (4 May- 27 June 2019 and 20 May- 2 July 2021) and late season (11 July-15 August 2019 and 07 July-25 August 2021) separately. Oleander aphids (*Aphis nerii*) were by far the most abundant herbivores found on milkweed plants, and the cumulative number of aphids was compared between common milkweed and swamp milkweed using a negative binomial generalized linear model (GLM) since the data did not fit the normal distribution. Data were analyzed using statistical software R (ver. 4.2.2) [34].

### Influence of local patch features and landscape context on monarch egg abundance

We used structural equation modeling (SEM) to examine how multiple variables associated with the habitat, landscape and insect community interacted to determine monarch egg abundance on milkweeds. Separate SEM models were run for common milkweed and swamp milkweed for both 2019 and 2021 due to phenological differences in monarch egg abundance and aphid colonization between milkweed species. The landscape-level treatments of location (abutting a wooded tree line or in an open crop field) and plant community (milkweed planted within a diverse wildflower matrix or monoculture tall fescue matrix) were included as binary categorical/state exogenous variables. Tree line is the reference state for location used in the model; therefore, a significant effect of tree line indicates significant difference from the open field crop habitat (the alternative state). Likewise, wildflower presence is the reference plant community state and significance indicates difference from the fescue monoculture. Endogenous variables included counts of predatory lady beetle adults and larvae, oleander aphids and monarch eggs. Lady beetles (Coccinellidae) are documented predators of monarch eggs [20,21] and were the most abundant generalist predators observed on milkweed plants. Oleander aphids were the most abundant and widespread herbivore across all milkweed plots.

**Fig 1:**
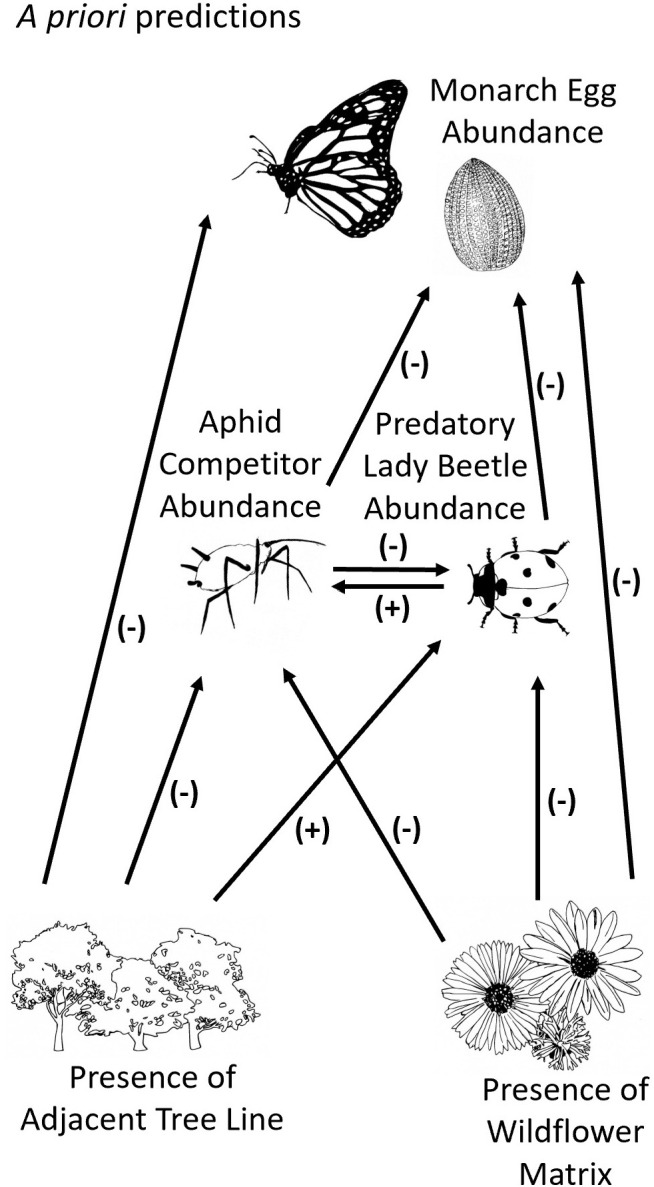
*A priori* predictions. *A priori* predictions of the direct effects among monarch eggs, oleander aphids, lady beetles, tree line location (as opposed to open crop environment) and diverse wildflower matrix (as opposed to fescue monoculture) used to create the structural equation model.

SEM analysis was based on *a priori* predictions of relationships among factors (Fig 1). We predicted that the presence of a diverse wildflower matrix as opposed to fescue monoculture would negatively affect monarch egg abundance. Given that adult monarch butterflies had access to flight fuel from wildflowers present in the wider surrounding landscape, we predicted that the main effect of the heterogenous mixture of wildflowers immediately surrounding milkweed plants would be to obscure chemical or visual signals, making the host plants more difficult to locate [29,30]. We also predicted that milkweed plots adjacent to the tree line would have a lower abundance of monarch eggs than plots in open crop environments because of the use of insecticides in conventionally managed agriculture and increased pressure of predators immigrating from the trees [33]. We predicted that presence of trees would have a positive effect on lady beetle abundance, since lady beetles sometimes aggregate to tree lines [33] and a negative effect on oleander aphids due to higher predation pressure. Wildflower matrix was predicted to have a negative direct effect on lady beetles as compared to surrounding fescue, since it would be more difficult for lady beetles to find their aphid prey within the heterogenous mixture [29,30,35]. The presence of flowering plants surrounding a target plant reduces aphid colonization, therefore we predicted lower aphid abundances among milkweed within the wildflower matrix [36]. We predicted that lady beetles and oleander aphids would negatively affect monarch egg abundance through predation (lady beetles), competition with female adult monarchs for milkweed plant resources like nectar and leaf surface area for oviposition (oleander aphids), and reduction of disease fighting foliar cardenolides (oleander aphids) [18,20], as well as through apparent competition (i.e., aphids attracting generalist lady beetle predators that also consume monarch eggs) [24]. Finally, we predicted that lady beetles would form a numerical response to their aphid prey [20].

The *a priori* predictions were translated into three regression equations, one predicting lady beetle abundance, one predicting aphid abundance and one predicting monarch egg abundance, and the SEM was constructed by integrating the individual regression models. The SEM was analyzed using *piecewise* SEM and the partial regression coefficients associated with each explanatory factor represent the strength of the direct effects. A negative binomial correction was used because the count data were non-normal and over dispersed, meaning the variance of the data is greater than the mean for all three response variables. Links that were not significant were removed from the final models. Data were analyzed using statistical software R (ver. 4.2.2) with packages “piecewisesem” and “MASS” [37,38]. The SEM models include observations recorded from July 11^th^ through August 15^th^ in 2019 and from July 7^th^ through August 25^th^ in 2021. This is the timeframe when aphids were present, but before plant quality began to decline at the end of the season.

Wildflowers in the surrounding matrix reached heights up to twice that of the focal milkweed plants (S1 Appendix). To assess the potential effect of plant-plant competition on milkweed growth in the plots with a diverse wildflower matrix, the average heights of swamp milkweed and common milkweed were compared in the presence and absence of wildflowers by running Wilcoxon-rank sum tests due to non-normality of the data.

## Results

### Comparison of monarch eggs and aphids across milkweed species

**Fig 2:**
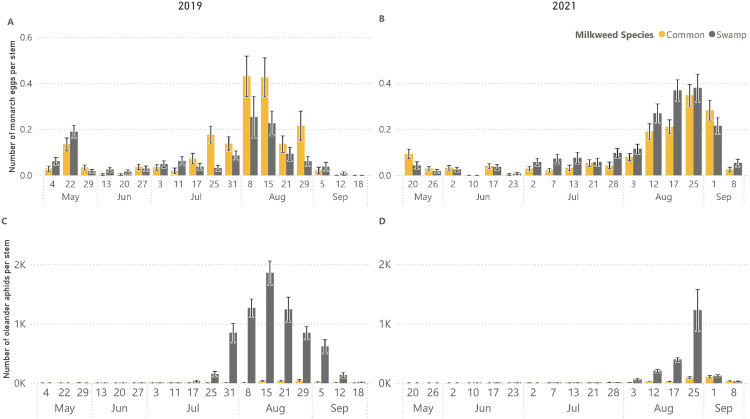
Monarch egg abundance and oleander aphid abundance. **A**) Monarch egg abundance per stem in 2019 for common milkweed (*Asclepias syriaca*) and swamp milkweed (*Asclepias incarnata*). Monarch egg abundance in the early season (4 May 2019−27 June 2019) was higher on swamp milkweed than common milkweed (p < 0.05). Late in the season (11 July 2019−15 August 2019), monarch egg abundance was higher on common milkweed than swamp milkweed (p < 0.0001). **B)** Monarch egg abundance per stem in 2021 for common milkweed and swamp milkweed. There was no statistical difference between monarch egg abundance on swamp milkweed vs. common milkweed early in the season (20 May 2021−2 July 2021) (p = 0.72). Late in the season (7 July 2021−25 August 2021), there were significantly more monarch eggs on swamp milkweed than on common milkweed (p < 0.05). **C**) Oleander aphid (*Aphis nerii*) abundance per stem in 2019 for common milkweed and swamp milkweed. Significantly more aphids were present on swamp milkweed than on common milkweed (p < 0.0001). **D)** Oleander aphid abundance per stem in 2021 for common milkweed and swamp milkweed. Significantly more aphids were present on swamp milkweed than on common milkweed (p < 0.0001). Mean ± SEM shown. Common milkweed (*Asclepias syriaca*) = gold bars, swamp milkweed (*Asclepias incarnata*) = gray bars.

Cumulatively across the 2019 season, more monarch eggs were found on common milkweed than swamp milkweed (common milkweed total eggs = 291, swamp milkweed total eggs = 236, *p* < 0.01, Fig 2 A). However, monarch egg abundance was bimodal, with a smaller early season peak and larger late-summer peak. In the early season, more monarch eggs were found on swamp milkweed than on common (*p* < 0.05). In the late season, there were significantly more monarch eggs on common milkweed than on swamp milkweed (*p* < 0.001).

Cumulatively in 2021, more monarch eggs were found on swamp milkweed than on common milkweed (common milkweed total eggs = 422, swamp milkweed total eggs = 527, p < 0.01) (Fig 2 B). In the early season of 2021, there was not a significant difference in monarch egg abundance between the milkweed species (*p* = 0.34); however, there were significantly more monarch eggs on swamp milkweed than on common milkweed late in the season (*p* < 0.001).

Oleander aphids were found on both common milkweed and swamp milkweed but were noticeably more abundant on swamp milkweed in both years (*p* < 0.0001) (Figs 2 C and D). Aphids appeared on milkweed plants in early July. The number of aphids increased exponentially on swamp milkweed, reaching a peak of more than 1,800 aphids per plant in the middle of August 2019 and more than 1,200 in 2021. Aphid abundance on common milkweed remained low throughout the summer, barely exceeding an average of 100 aphids per plant in 2019 and never exceeding 50 aphids per plant in 2021.

### Influence of local patch features and landscape context on monarch egg abundance

#### Swamp milkweed.

**Fig 3:**
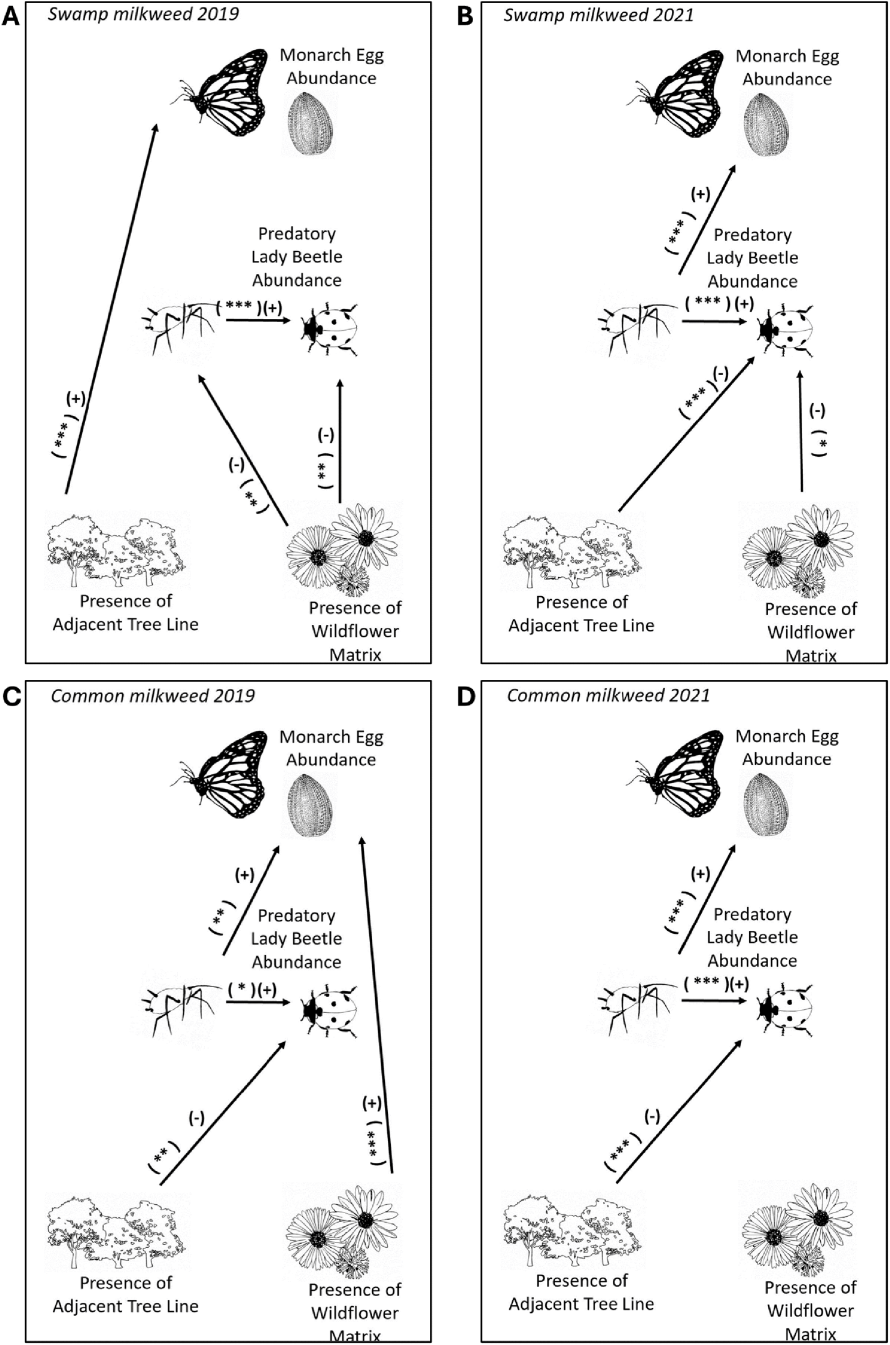
Results of structural equation model asking how patch characteristics and landscape context affect monarch egg abundance. **A)** On swamp milkweed (*Asclepias incarnata*) in 2019 and **B)** in 2021. **C)** On common milkweed (*Asclepias syriaca*) in 2019 and **D)** in 2021. Each arrow represents a significant (+) positive or (-) negative relationship. Stars indicate level of significance: p < 0.1*, p < 0.05**, and p < 0.01***.

Patch features and landscape context directly affected monarch egg abundance on swamp milkweed, but the responses differed in 2019 and 2021 (Figs 3 A and B). At the patch level, monarch eggs were positively associated with aphid abundance in 2021 (*p* < 0.0001, *coefficient* = 0.15) but had no association in 2019. Lady beetles formed a numerical response to aphid prey, resulting in a positive association between lady beetles and aphids in both years (2019: *p* = 0.0001, *coefficient* = 0.18; 2021: *p* < 0.0001, *coefficient* = 0.37). At the landscape level, the presence of a tree line had a positive direct effect on monarch eggs as compared to the open crop environment in 2019 (*p* < 0.0001, *coefficient* = 1.51). There were no direct landscape-level effects on monarch egg abundance in 2021. Lady beetle abundance was lower in plots adjacent to tree lines than those in the open crop environment in 2021 (p < 0.0001, *coefficient* = −1.78). The presence of surrounding wildflower matrix had a negative effect on lady beetle abundance as compared to surrounding fescue monoculture in both years (2019: *p* < 0.05, *coefficient* = −0.78; 2021: *p* < 0.1, *coefficient* = −0.29) and a negative effect on aphid abundance in 2019 (*p* < 0.05, *coefficient* = −0.53). The presence of a wildflower matrix decreased the height of swamp milkweed plants (*p* < 0.001, average milkweed plant height in wildflower matrix = 40.2 ± 1.026 cm and fescue matrix = 46.9 ± 1.098 cm, mean ± SEM).

#### Common milkweed.

Patch-level features directly affected monarch egg abundance on common milkweed in 2019 and 2021 (Figs 3 C and D). Monarch egg abundance was positively related to aphid abundance in both years (2019: *p* < 0.05, *coefficient* = 0.17; 2021: *p* < 0.0001, *coefficient* = 0.0018). Lady beetles formed a numerical response to aphid prey, resulting in a positive association between lady beetles and aphids in both years (2019: *p* < 0.1, *coefficient* = 0.27; 2021: *p* < 0.001, *coefficient* = 0.21). There was limited evidence that landscape context influenced the abundance of monarch eggs on common milkweed. The presence of a wildflower matrix had a positive effect on monarch eggs as compared to surrounding fescue monoculture in 2019 (*p* < 0.01, *coefficient* = 0.54). Lady beetle abundance was lower in plots adjacent to tree lines than those in the open crop environment in both years (2019: *p* < 0.05, *coefficient* = −1.32; 2021: *p* < 0.0001, *coefficient* = −1.99). The height of common milkweed plants was not affected by surrounding wildflower matrix (*p* = 0.28).

## Discussion

New milkweed habitats are being constructed to support diminished monarch butterfly populations [1,2,5]. The goal is to design monarch habitats that optimize access to required plant resources while minimizing negative interactions. In this study, we examined the effects of local patch features and landscape context on the quality of habitat for monarchs in an agricultural environment. We found that patch characteristics had the most significant effects on monarch egg abundance on milkweed plants. Specifically, milkweed species identity and the presence of co-occurring insects played an important role in determining the number of monarch eggs on plants, while the landscape effects of wildflowers and the proximity to wooded areas were less important. The challenge is that the exact nature of these relationships varied over time, complicating habitat planting recommendations.

At the patch level, milkweed species identity was an important determinant of monarch egg abundance, but the responses differed across years. In 2019 we found that there were more monarch eggs on common milkweed than swamp milkweed cumulatively over the season. This result is contrary to previous controlled environment studies that documented greater monarch preference for and performance on swamp milkweed [9–13]. However, it is notable that there were two peaks in egg abundance over time, and the milkweed species with the most eggs flipped from swamp milkweed early season to common milkweed late season. Early season egg laying in the Midwest United States occurs as overwintering adults from Mexico migrate north [39]. At this point in the 2019 season, egg abundance was higher on swamp milkweed than common milkweed, but the overall abundance of eggs was comparatively low as breeding females and subsequent generations continued to migrate north throughout spring and early summer. Late in the 2019 season when reproduction by breeding populations of monarchs is high [39,40], we observed the opposite pattern of more monarch eggs on common milkweed than swamp milkweed. This unexpected result is attributed to the open nature of our field study where uncontrolled factors, such as plant phenology, competitor-mediated changes to host plant quality, predation, or other abiotic and biotic forces, may confound monarch oviposition preferences and egg performance on different milkweed species [20–22,41]. Another important difference is that controlled studies measure oviposition directly while we quantified the number of surviving monarch eggs on plants, which is a function of both the female’s oviposition decision and egg survival to larval emergence. In 2021 we found the opposite result, with cumulative egg abundance being higher on swamp milkweed than common milkweed as predicted by previous studies [9,11,13]. Notably, overall oleander aphid abundance was lower in 2021 than 2019, potentially reducing the confounding influences of co-occurring insects in that year. While the exact mechanism is not known, it is clear that monarchs respond differently to different milkweed species and that the fluctuating environmental context in which milkweed habitats are established influences their value for monarchs over time, emphasizing the need for real-world multi-year host preference and performance studies to complement controlled studies in the greenhouse and lab.

Oleander aphid abundance had the most consistent effect on monarch eggs of any measured factor. We detected a positive association between aphid abundance and monarch egg abundance on both milkweed species, despite significantly higher aphid abundance on swamp milkweed than common milkweed. A positive association between monarch eggs and oleander aphids was surprising since these aphids can act as competitors of adult and larval monarchs, consuming plant phloem, occupying leaf surface area, and decreasing plant quality through direct usage of plant resources [42]. This positive association may be an artifact of the temporal overlap in the phenology of monarch and aphid populations, which both build in numbers late in the season. However, time was incorporated into our model and there is experimental support for a direct causal relationship. Researchers have found that aphid feeding can increase host plant quality for monarch caterpillars due to crosstalk in defensive pathways [17] and an increase in the availability of free amino acids in the plant tissue [43]. Adult female monarchs may sense these chemical changes associated with plant quality and alter their oviposition behavior accordingly. In fact, we demonstrated in a previous greenhouse study that monarch egg deposition is an increasing function of the number of aphids on a plant (up to moderately high densities of aphids) [44]. Predation may also play a role in the positive relationship between monarch eggs and oleander aphids. Although natural enemy mediated apparent competition is common in insect communities [45], and we predicted it here, we did not detect evidence of its occurrence. Lady beetle predators were attracted to plants with aphids, but the presence of more generalist predators on milkweed plants did not result in the suppression of monarch eggs. Instead, it appears that oleander aphids served as an alternative prey for lady beetles, indirectly reducing the consumption of monarch eggs in the field despite higher numbers of predators. Overall, while it is likely that aphid co-occurrence on milkweed plants is an important environmental factor directly influencing the quality of habitats for monarchs, more study on the potential direct and indirect causal relationships between aphids and monarch eggs is needed.

Landscape context also affected monarch egg abundance, but the particular factors involved differed between the two species of milkweed and over time. Trees were important contributors to egg abundance on swamp milkweed in 2019. Swamp milkweed abutting wooded areas hosted more monarch eggs than swamp milkweed located in the grassy edge strips between soybean and maize fields. Notably, trees had no effect on monarch eggs in 2021. Columbia, MO experienced a flash drought in July 2019, with rainfall totals for July half of that received in 2021. During this time, soil adjacent to wooded areas was drier than crop edges due to water use by trees, creating an environment with limited water availability for milkweeds. Swamp milkweed, as its name implies, prefers moist to wet soil [46]. It is possible that water-limited swamp milkweed plants experienced osmotic stress, interfering with plant defense responses and making nitrogen more available throughout the plant tissue for insects [47]. Water stressed plants with fewer defenses and higher in available nitrogen may have been more attractive to ovipositing monarchs [48,49]. It would be interesting to explore the effects of yearly variations in weather on habitat quality for monarchs, but more data are needed as this study was limited to only two years. Another potential explanation for monarch eggs benefitting from the presence of trees is that adult butterflies may have used wooded habitat for protection from predators or as an escape from the hot sun [32]. We often observed adult monarchs resting in the foliage of the trees, and it is possible that ovipositing females using this habitat would lay eggs in adjacent plots more frequently than those in the open crop environment. If this is the case, however, it is unclear why this benefit of proximity to trees was not detected in 2021 or on common milkweed.

The wildflower matrix was an important determinant of egg abundance on common milkweed, but only in 2019. We found a larger number of monarch eggs on common milkweed when embedded within a diverse wildflower matrix than when surrounded by a monoculture of tall fescue. This result is consistent with previous research, which found that 22% more eggs were laid on milkweed planted in mixed species plots as opposed to a monoculture [27]. Common milkweed blooms early in the summer, with flowering completed by late June. Wildflowers may serve as an important source of nectar for breeding generation females ovipositing on non-flowering common milkweed later in the summer. Interestingly, wildflowers did not affect egg abundance on swamp milkweed, which blooms later in the season and for a longer duration. These results also suggest that ovipositing monarchs can still locate their host plants among a heterogeneous mix of wildflowers and that plant apparency is not a significant issue affecting their ability to locate milkweed resources [50].

Landscape context also influenced the composition of the insect community co-occurring on milkweed plants. Lady beetles were negatively affected when common milkweed was located in close proximity to trees as compared to the grassy margins of soybean and maize fields. This result was unexpected since we hypothesized that wooded areas would act as a refuge for predators [33]. It is possible that lady beetles either prefer open habitats or are better able to track prey in open habitats. Complex habitat structure was previously shown to interfere with the search behavior and numerical response of lady beetles [51]. Similarly, we found that the wildflower matrix had a negative effect on lady beetle abundance on swamp milkweed plants in 2019 and 2021. Wildflowers may have served as an additional source of prey for lady beetles, drawing lady beetles away from the milkweed plants. Wildflowers may also interfere with the ability of lady beetles to locate milkweed host plants. Swamp milkweed plants grown in association with relatively tall wildflowers were smaller than those with surrounding grass. Furthermore, a heterogeneous mix of vegetation often makes finding target plants more difficult by creating physical barriers to seeing plants or by masking chemicals released by host plants [30]. Lady beetles searching for aphids on milkweed plants may have a more difficult time locating the prey in plots with more vegetation since lady beetles are known to respond to aphid induced plant volatiles [52] and these volatiles may be harder to isolate in a diverse habitat. Additionally, habitat complexity also likely influenced their prey searching by increasing search time through changes in rate of search [35].

## Conclusions

Optimizing habitat for monarchs is key to mitigating their decline. Assessing how patch- and landscape-level factors affect the quality of monarch habitats in agricultural environments, where the availability of milkweed has declined sharply over the past two decades, is particularly important knowledge to design successful habitats for conservation. The results of this study can inform land managers tasked with designing and prioritizing critical conservation habitat plantings for this at-risk species, but with the caveat that additional field studies across a diversity of environmental contexts and years are necessary before final recommendations can be made. Our work suggests that local patch characteristics have the most consistent effects on monarch egg abundance, which is encouraging because it can be easier to control how habitats are created and managed than it is to control where the land is available to create habitats. Our work suggests that monarchs benefit from including more than one milkweed species in a habitat planting as insurance against fluctuating biotic and abiotic conditions. Since oleander aphids may positively affect monarch eggs, direct management of these insects is likely not required, unless they risk killing the plant. Including wildflowers in the landscape as pollinator nectar resources can increase monarch egg abundance without interfering with host plant location by monarchs. And finally, monarch habitats should be planted in close proximity to wooded areas where possible.

## Supporting information

S1 Appendix Photo of plot with surrounding wildflower matrix. An example plot of milkweed located within a diverse mix of wildflower species.
(JPG)

S2 Appendix Photo of plot with surrounding fescue monoculture. An example plot of milkweed surrounded by a monoculture of tall fescue grass (Festuca arundinacea).
(JPG)

S3 Appendix Stylized example of one block (n = 10) of the field plot design. Each block consisted of four plots containing 14 milkweed plants each, either swamp (Asclepias incarnata) or common (Asclepias syriaca). Each milkweed species treatment was crossed with a plant diversity treatment (10 species of wildflower nectar plants or a monoculture of tall fescue grass surrounding the milkweed) for a total of four treatments.
(JPG)

S4 Appendix Photo of plot adjacent to trees. Flagged location of a block placement located within 5m of a tree line.
(JPG)

S5 Appendix Photos of plot in open field crop environment. Location of an open field block surrounded by soybean (Glycine max) and maize crops (Zea mays).
(JPG)

S6 Appendix Wildflower plant species (codes 1–10) used in plot establishment. Each species was chosen based on bloom phenology and hardiness to Missouri climate. Species were also chosen to vary in bloom color.
(JPG)

S7 Appendix Example layout for an experimental plot containing wildflowers. Twenty-eight seedling plugs of each of ten native wildflower species (280 total) were planted. The location of each seedling around the center milkweed patch (indicated in dark green) was randomly assigned using a random number generator. Numbers correspond to the identity of wildflower species assigned to each location based on S6 Appendix.
(JPG)

S8 Appendix R code. (DOCX)

S1 Dataset Raw data. (XLSX)
