# Supplementary figures and images for: Context-dependency of monarch butterfly (Danaus plexippus) egg abundance on milkweeds (Asclepias)
Source: PLoS One. 2025 Nov 4;20(11):e0336242. doi: 10.1371/journal.pone.0336242 (PMC12585044; doi:10.1371/journal.pone.0336242)

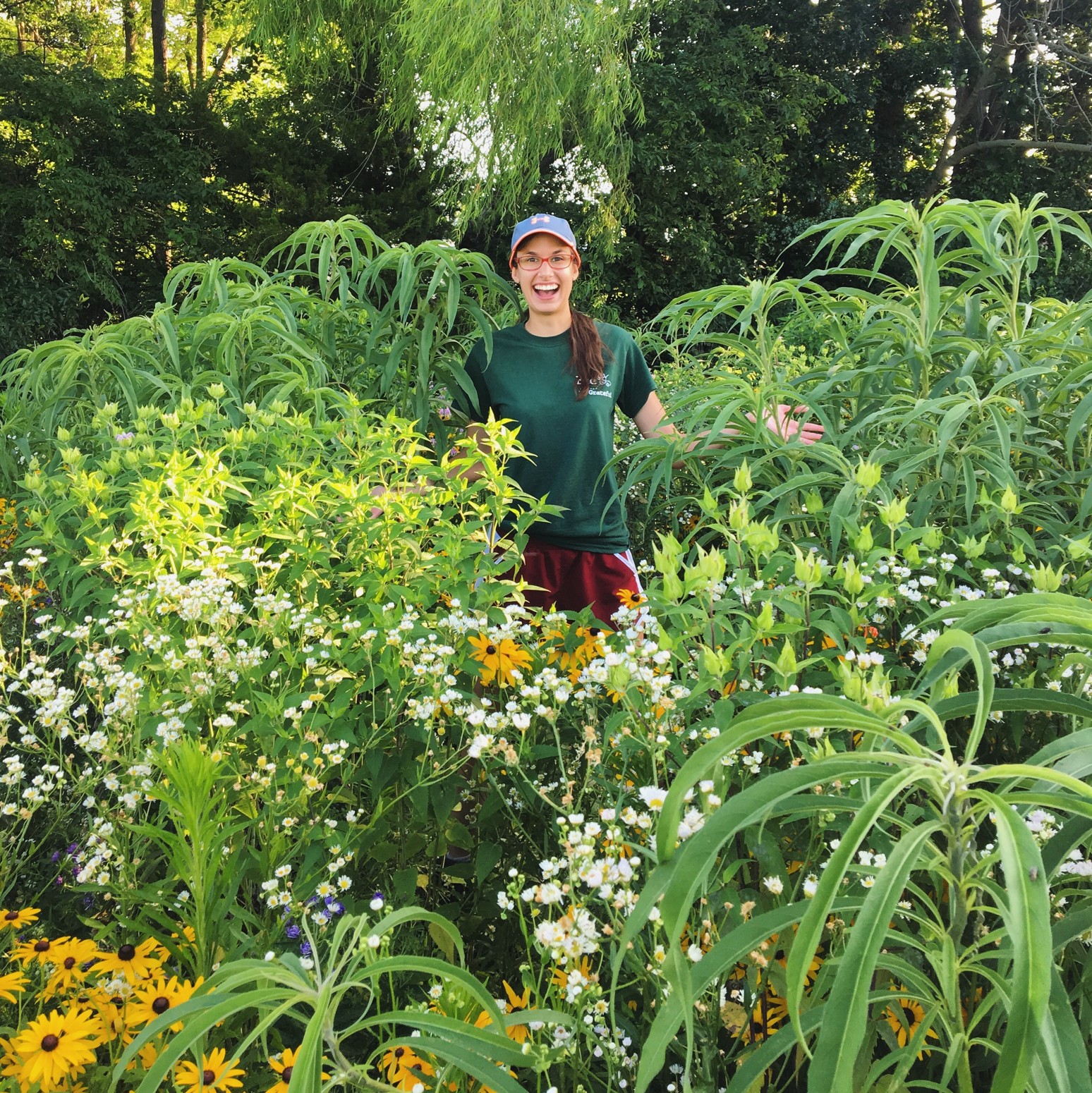

Supplement: S1 Appendix — An example plot of milkweed located within a diverse mix of wildflower species. (JPG) [file pone.0336242.s001.jpg]

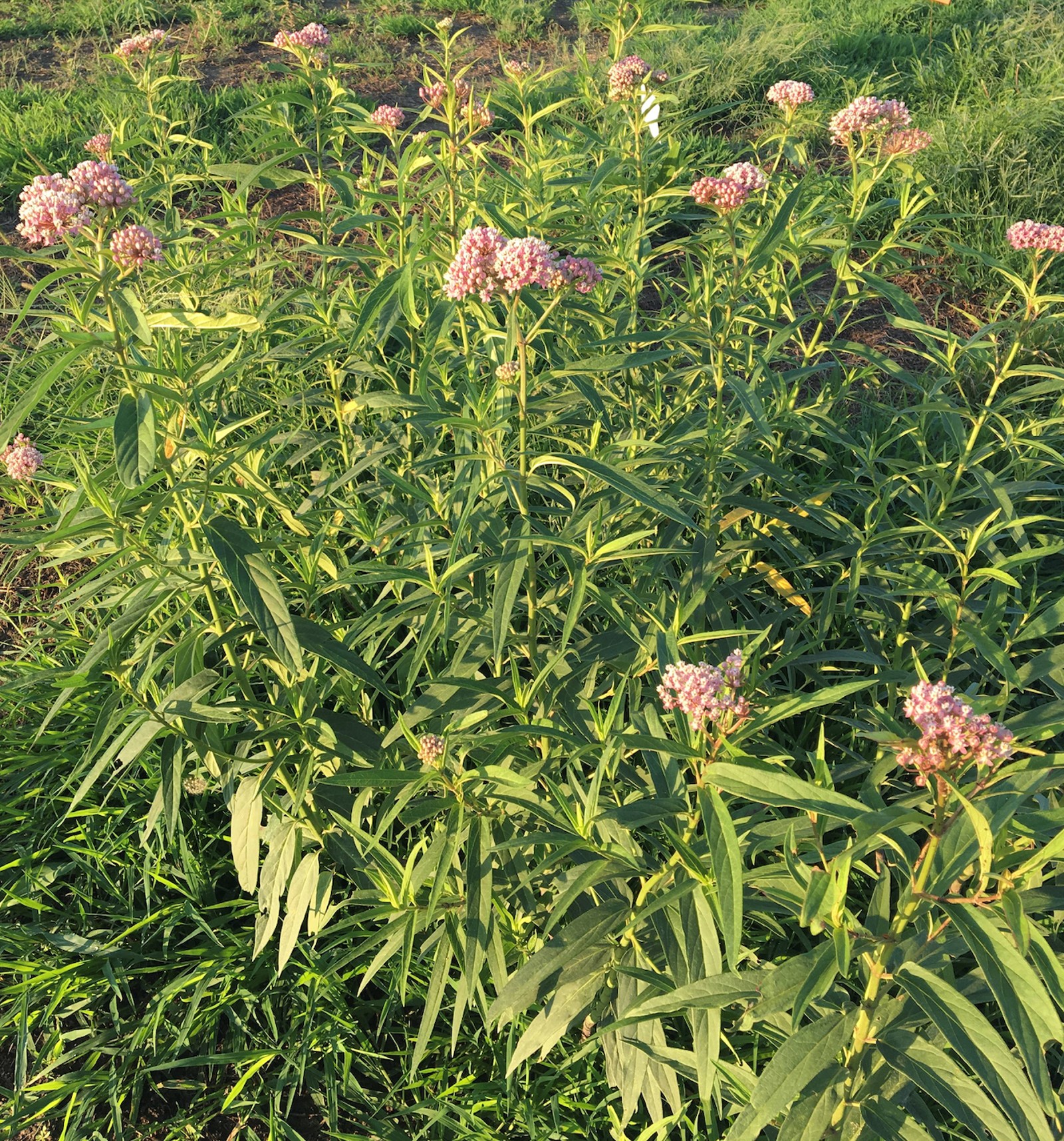

Supplement: S2 Appendix — An example plot of milkweed surrounded by a monoculture of tall fescue grass (Festuca arundinacea). (JPG) [file pone.0336242.s002.jpg]

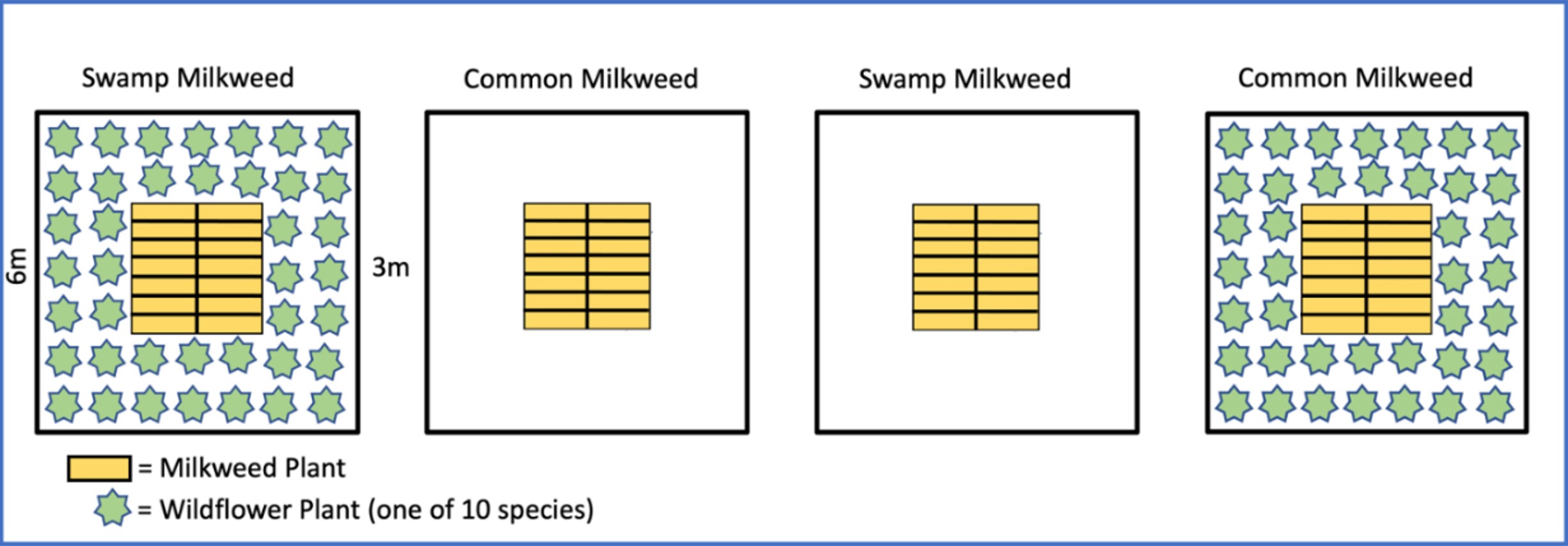

Supplement: S3 Appendix — Each block consisted of four plots containing 14 milkweed plants each, either swamp (Asclepias incarnata) or common (Asclepias syriaca). Each milkweed species treatment was crossed with a plant diversity treatment (10 species of wildflower nectar plants or a monoculture of tall fescue grass surrounding the milkweed) for a total of four treatments. (JPG) [file pone.0336242.s003.jpg]

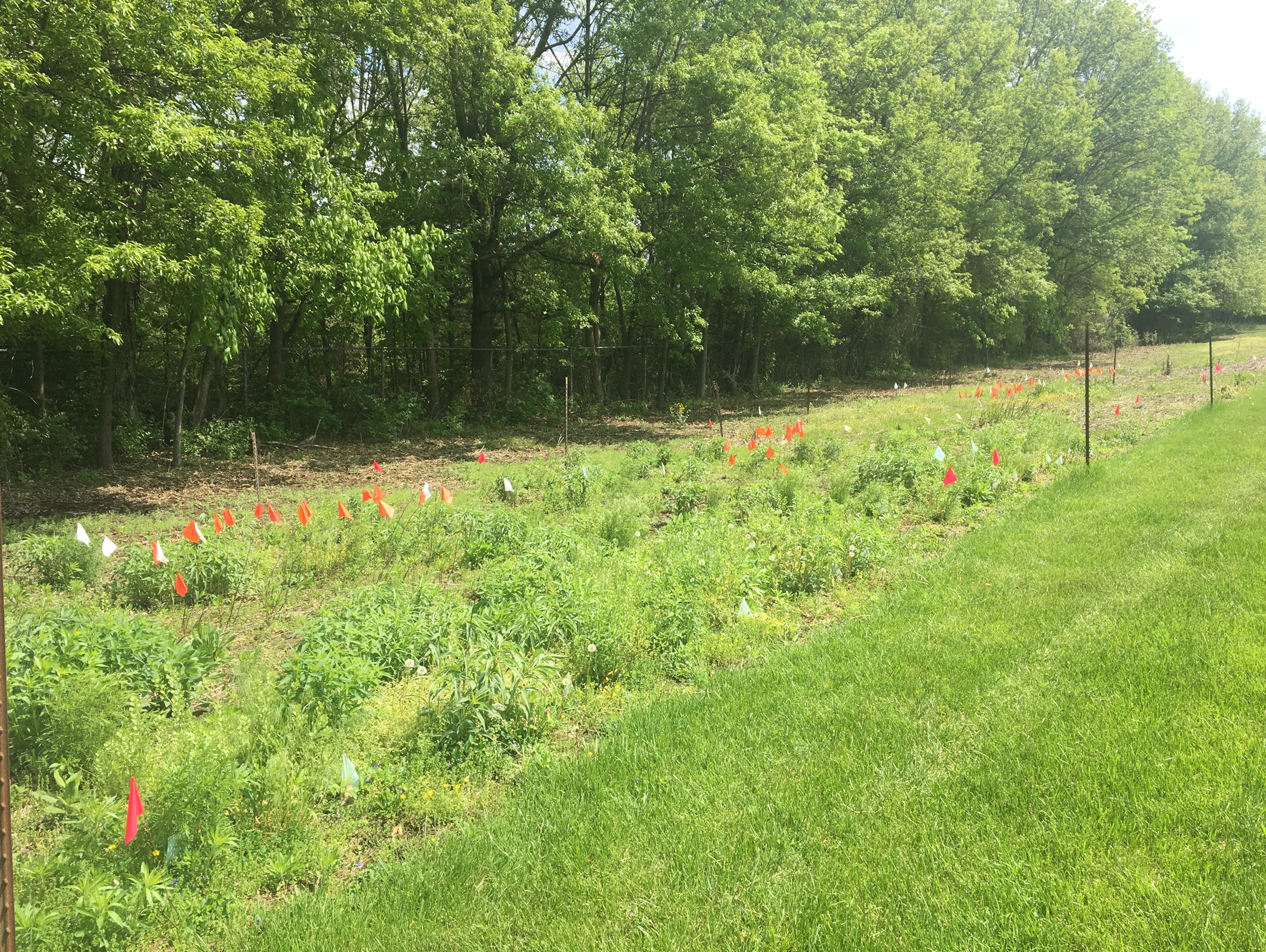

Supplement: S4 Appendix — Flagged location of a block placement located within 5m of a tree line. (JPG) [file pone.0336242.s004.jpg]

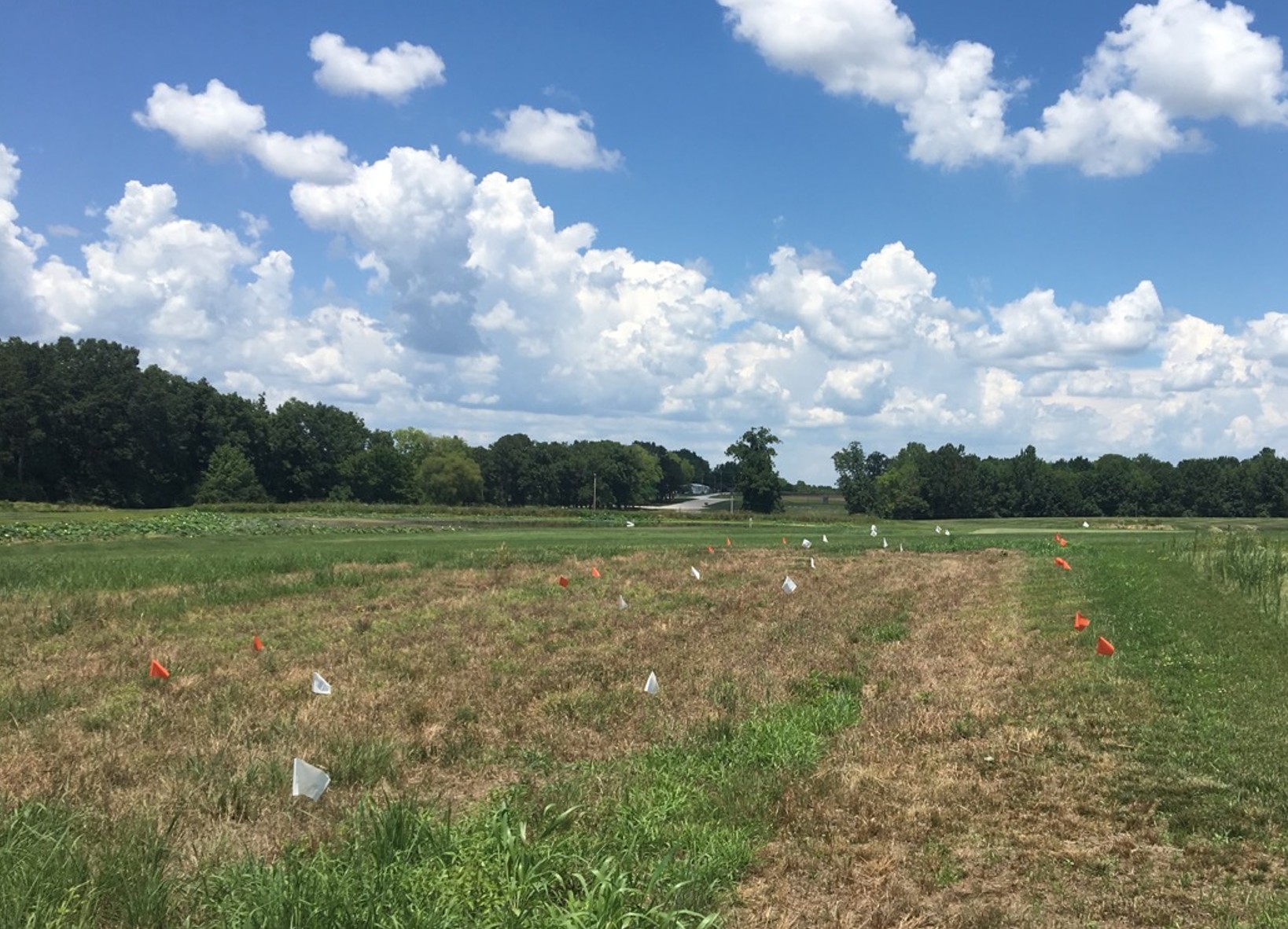

Supplement: S5 Appendix — Location of an open field block surrounded by soybean (Glycine max) and maize crops (Zea mays). (JPG) [file pone.0336242.s005.jpg]

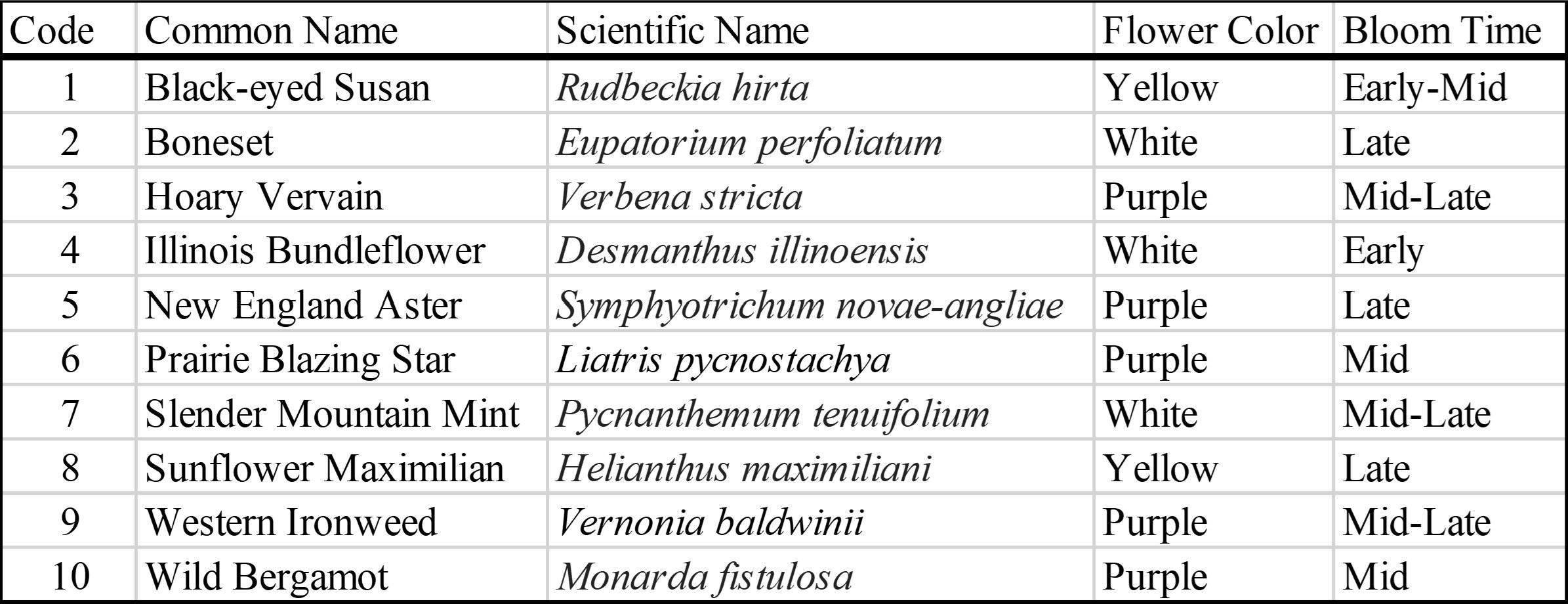

Supplement: S6 Appendix — Each species was chosen based on bloom phenology and hardiness to Missouri climate. Species were also chosen to vary in bloom color. (JPG) [file pone.0336242.s006.jpg]

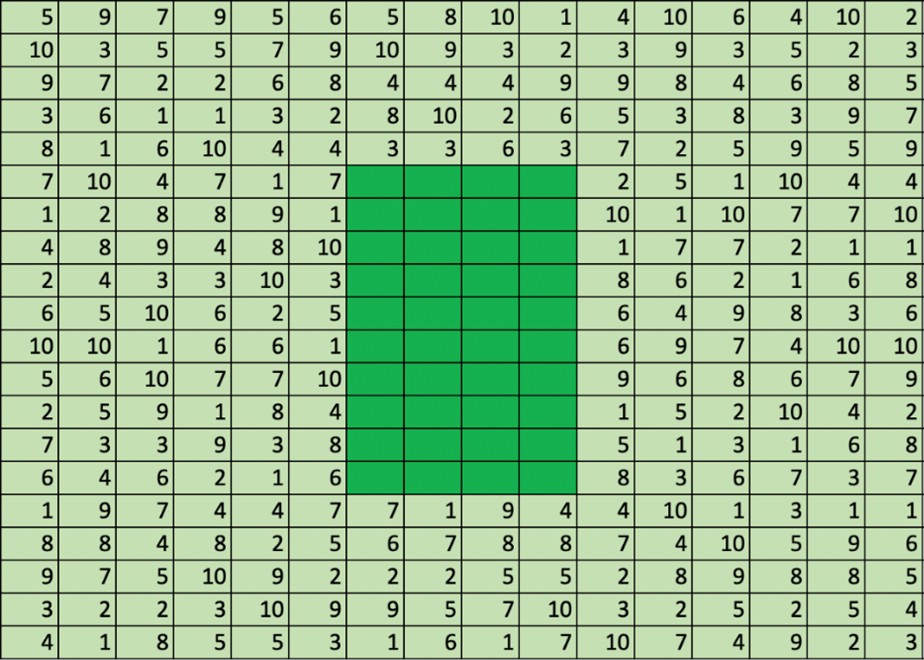

Supplement: S7 Appendix — Twenty-eight seedling plugs of each of ten native wildflower species (280 total) were planted. The location of each seedling around the center milkweed patch (indicated in dark green) was randomly assigned using a random number generator. Numbers correspond to the identity of wildflower species assigned to each location based on S6 Appendix. (JPG) [file pone.0336242.s007.jpg]
