# Supplementary material for: Context-dependency of monarch butterfly (Danaus plexippus) egg abundance on milkweeds (Asclepias)
Source: PLoS One. 2025 Nov 4;20(11):e0336242. doi: 10.1371/journal.pone.0336242 (PMC12585044; doi:10.1371/journal.pone.0336242)
Supplement: S8 Appendix — (DOCX) [file pone.0336242.s008.docx]

df2= read.csv('C:/Monarch Data 2019 for Analysis.csv')

df2$Opene <- as.factor(df2$Open)

df2$plotID <- as.factor(df2$plotID)

df2$Week <- as.factor(df2$Week)

df2$Plot <- as.factor(df2$Plot)

df2$Block <- as.factor(df2$Block)

df2$logaphids<- log1p(df2$aphids)

#Creating models for relevant dates of 2019 season for Swamp milkweed plots

df3<- df2[which(df2$SwampCommon == 'Swamp'),]

dfl <- df3[which(df3$Week %in% c( '7/11/2019', '7/17/2019', '7/25/2019', '7/31/2019', '8/1/2019', '8/8/2019', '8/15/2019')),]

library(glmmTMB)

swamp_2019_aphid_model<- glmmTMB::glmmTMB(aphids~Opene+ Nectar + (1|Block/Plot) + (1|Week) ,family = nbinom2,data = dfl)

swamp_2019_egg_model <- glmmTMB::glmmTMB(Eggs~Opene+ Nectar + logaphids + (1|Block/Plot)+ (1|Week) , family = nbinom2,data = dfl, control = glmmTMBControl(optimizer = optim,

optArgs = list(method = "BFGS")))###changed optimizer for convergence

swamp_2019_ladybeetle_model <- glmmTMB::glmmTMB(LadyBeetles~Opene+ Nectar + logaphids + (1|Block/Plot)+ (1|Week), family = nbinom2,data = dfl)

summary(swamp_2019_aphid_model)

summary(swamp_2019_egg_model)

summary(swamp_2019_ladybeetle_model)

library(pscl)

pR2(swamp_2019_aphid_model)

pR2(swamp_2019_egg_model)

pR2(swamp_2019_ladybeetle_model)

#Creating models for relevant dates of 2019 season for Common milkweed plots

df4<- df2[which(df2$SwampCommon == 'Common'),]

dflc <- df4[which(df4$Week %in% c( '7/11/2019', '7/17/2019', '7/25/2019', '7/31/2019', '8/1/2019', '8/8/2019', '8/15/2019')),]

common_2019_aphid_model<- glmmTMB::glmmTMB(aphids~Opene+ Nectar + (1|Block/Plot) + (1|Week) ,family = nbinom2, data = dflc)

common_2019_egg_model <- glmmTMB::glmmTMB(Eggs~Opene+ Nectar + logaphids + (1|Block/Plot)+ (1|Week) , family = nbinom2,data = dflc)

common_2019_ladybeetle_model <- glmmTMB::glmmTMB(LadyBeetles~Opene+ Nectar + logaphids + (1|Block/Plot)+ (1|Week), family = nbinom2,data = dflc)

summary(common_2019_aphid_model)

summary(common_2019_egg_model)

summary(common_2019_ladybeetle_model)

pR2(common_2019_aphid_model)

pR2(common_2019_egg_model)

pR2(common_2019_ladybeetle_model)

## Models are too complex for PSEM, use the model summaries to generate SEM / path analysis manually

#Overall 2019 season, were there more aphids/Eggs on swamp or common milkweed?

m0 <- glm.nb(df2$Eggs ~ df2$SwampCommon, data = df2)

summary(m0)

m0x <- glm.nb(df2$aphids ~ df2$SwampCommon, data = df2)

summary(m0x)

#What about late season?

dfl <- df2[which(df2$Week %in% c( '7/11/2019', '7/17/2019', '7/25/2019', '7/31/2019', '8/1/2019', '8/8/2019', '8/15/2019')),]

m1 <- glm.nb(dfl$Eggs ~ dfl$SwampCommon, data = dfl)

summary(m1)

#What about early season?

dfl <- df2[which(df2$Week %in% c( '5/4/2019', '5/22/2019', '5/29/2019', '6/13/2019', '6/20/2019', '6/27/2019', '7/3/2019')),]

m2 <- glm.nb(dfl$Eggs ~ dfl$SwampCommon, data = dfl)

summary(m2)

## Onto 2021 season data

df21= read.csv('C:/Monarch Data 2021 for Analysis.csv')

df21$logaphids<- log1p(df21$aphids)

df21$Plot <- as.factor(df21$Plot)

df21$Block <- as.factor(df21$Block)

df21$Week <- as.factor(df21$Date)

#Creating models for relevant dates of 2021 season for Common milkweed plots

df21x<- df21[which(df21$SwampCommon == 'Common'),]

df21x <- df21x[which(

df21x$Date == '2021-07-07' |

df21x$Date == '2021-07-13' |

df21x$Date == '2021-07-21' |

df21x$Date == '2021-07-28' |

df21x$Date == '2021-08-03' |

df21x$Date == '2021-08-12' |

df21x$Date == '2021-08-17' |

df21x$Date == '2021-08-25'

), ]

common_2021_aphid_model<- glmmTMB::glmmTMB(aphids~Opene+ Nectar + (1|Block/Plot) + (1|Week) ,family = nbinom2,data = df21x)#best

common_2021_egg_model <- glmmTMB::glmmTMB(Eggs~Opene+ Nectar + logaphids + (1|Block/Plot)+ (1|Week) , family = nbinom2,data = df21x)

common_2021_ladybeetle_model <- glmmTMB::glmmTMB(LadyBeetles~Opene+ Nectar + logaphids + (1|Block/Plot)+ (1|Week), family = nbinom2,data = df21x)

summary(common_2021_aphid_model)

summary(common_2021_egg_model)

summary(common_2021_ladybeetle_model)

pR2(common_2021_aphid_model)

pR2(common_2021_egg_model)

pR2(common_2021_ladybeetle_model)

#Creating models for relevant dates of 2021 season for Swamp milkweed plots

df21x<- df21[which(df21$SwampCommon == 'Swamp'),]

df21x <- df21x[which(

df21x$Date == '2021-07-07' |

df21x$Date == '2021-07-13' |

df21x$Date == '2021-07-21' |

df21x$Date == '2021-07-28' |

df21x$Date == '2021-08-03' |

df21x$Date == '2021-08-12' |

df21x$Date == '2021-08-17' |

df21x$Date == '2021-08-25'

), ]

swamp_2021_aphid_model<- glmmTMB::glmmTMB(aphids~Opene+ Nectar + (1|Block/Plot) + (1|Week) ,family = nbinom2,data = df21x)#best

swamp_2021_egg_model <- glmmTMB::glmmTMB(Eggs~Opene+ Nectar + logaphids + (1|Block/Plot)+ (1|Week) , family = nbinom2,data = df21x)

swamp_2021_ladybeetle_model <- glmmTMB::glmmTMB(LadyBeetles~Opene+ Nectar + logaphids + (1|Block/Plot)+ (1|Week), family = nbinom2,data = df21x)

summary(swamp_2021_aphid_model)

summary(swamp_2021_egg_model)

summary(swamp_2021_ladybeetle_model)

pR2(swamp_2021_aphid_model)

pR2(swamp_2021_egg_model)

pR2(swamp_2021_ladybeetle_model)

## Models are too complex for PSEM, use the model summaries to generate SEM / path analysis manually

#Overall 2021 season, were there more aphids/Eggs on swamp or common milkweed?

m2 <- glm.nb(df21$Eggs ~ df21$SwampCommon, data = df21)

summary(m2)

m2 <- glm.nb(df21$aphids ~ df21$SwampCommon, data = df21)

summary(m2)

#What about late season 2021?

df21x <- df21[which(

df21$Date == '2021-07-07' |

df21$Date == '2021-07-13' |

df21$Date == '2021-07-21' |

df21$Date == '2021-07-28' |

df21$Date == '2021-08-03' |

df21$Date == '2021-08-12' |

df21$Date == '2021-08-17' |

df21$Date == '2021-08-25'

), ]

m2 <- glm.nb(df21x$Eggs ~ df21x$SwampCommon, data = df21x)

summary(m2)

#What about early season 2021?

df21x <- df21[which(df21$Date < '2021-07-07'), ]

m2 <- glm.nb(df21x$Eggs ~ df21x$SwampCommon, data = df21x)

summary(m2)
